# Platforms as laboratories of the social: How digital capitalism matters for computational social research in North America

**DOI:** 10.1177/03063127251321826

**Published:** 2025-02-25

**Authors:** Onurhan Ak

**Affiliations:** Department of Sociology, Queen’s University, Kingston, ON, Canada

**Keywords:** computational social science, epistemology, data science, machine learning

## Abstract

The contemporary prevalence of artificial intelligence and machine learning methods has resulted in a rich literature on the factors that shape computational research. This article draws on the laboratory studies literature to examine how platforms’ socio-technical infrastructures shape contemporary computational social science research. Based on 18 months of online ethnography of a university laboratory and 15 in-depth interviews with its researchers, the article makes two main arguments. First, for computational social sciences, platforms function as laboratories where the social is selectively carved and transformed, to make it knowable with computational methods. Thus, it makes the case that platforms manufacture the objects of analysis in computational social research and provide the social as a domain. Second, because of the significance of social media platforms as data laboratories for computational research, in contrast to the claims of data sciences to be domainless, these sciences may derive some of their epistemological and occupational power, as well as their cultural authority, from digital capitalism.

Drawing on 18 months of fieldwork in a university lab in North America, I show that platform-generated data plays an outsized role in contemporary computational social research because they tend to be more suitable for computational approaches than other data sources. Because of the different strategies the industry employs to standardize sociality, platform-generated data has a high degree of compatibility with computational methodologies. Thus, I claim that social media platforms act as laboratories that do prospecting work (Slota et al., 2020) and produce the social as a ‘domain’ (Ribes, 2020) that can be studied with computational methods. In other words, I argue that platforms fulfill the constitutive functions of laboratories and in doing so, they are performative. In addition, I have found that variations in platform designs can influence the choices of computational researchers when it comes to selecting a platform as a data source. These decisions are often based on how compatible the platform’s manufactured data are with their specific research questions.

I frame digital platforms as laboratories for computational social research. In this, (1) I depart from narratives that emphasize their economic or political agency, and (2) I highlight how the inner ‘technical’ workings and configurations of platforms have far-reaching epistemological consequences beyond political impacts for research that uses platform data. Second, by considering platforms as epistemological agents, I contribute to current debates about the proximity between academic research and Big Tech, especially with respect to Big Tech’s direct influence in shaping universities’ decisions and scholars’ research questions. In addition to its financial influence, that Big Tech influences and shapes academic research in epistemological and more indirect ways. These include Big Tech’s shaping of academic research through their de facto monopoly on data on social interactions, and also the ways in which platform data shapes what research questions are pursued in the first place.

Scholars have examined in some detail the politics of algorithms and data in terms of its consequences for social and economic inequities (Benjamin, 2019; Citron & Pasquale, 2014; Eubanks, 2017; Noble, 2018; Sweeney, 2013), along with the decision-making processes, practices, and cultures that inform the making of algorithms (Gillespie, 2014; Henriksen & Bechmann, 2020; Jaton, 2017, 2020; Seaver, 2019; Thakor, 2016). In addition, research on digital capitalism has paid much attention to the oppressive and violent nature of data extraction (Couldry & Mejias, 2019; Greene & Joseph, 2015; Sadowski, 2019, 2020; Srnicek, 2016; Thatcher et al., 2016) rather than its consequences for computational social epistemologies. While researchers have often pointed to the constructed nature of data on social media platforms (Couldry & Mejias, 2019; Dalton et al., 2016; Sadowski, 2019; Törnberg & Uitermark, 2021), the construction process has not been fully theoretically sketched out and its empirical implications remain an understudied dimension of how platforms shape contemporary knowledge production.

The connections between Big Tech’s and academia have also attracted attention. Researchers have raised concerns about the financial proximity of Big Tech and academia in computational fields (Abdalla & Abdalla, 2021), the alignment of academic research with public interests (Birhane et al., 2022; Stinson et al., 2020), and the influence of corporate needs over ethics (Phan et al., 2022). In this article I complement that work by looking at an understudied dimension of how platforms shape contemporary knowledge production, the epistemological influence of Big Tech in academic research.

## Platforms and the construction of ‘the social’

Many STS and other scholars have highlighted the constructed nature of data (Gitelman, 2013) and particularly of social media data (Kennedy, 2016). Scientists employ various strategies to construct data, such as embedding conceptual assumptions into simulations that generate data, as demonstrated in the development of modern economics (Brine & Poovey, 2013). Additionally, scholars have underlined that maintaining and ensuring the commensurability of data over time requires continuous care, immense infrastructure, and coordination (Ribes & Jackson, 2013). Furthermore, the literature has documented how the positionalities of data practitioners play roles in making data represent phenomena (Räsänen & Nyce, 2013; Whitman, 2020). Overall, there is no such thing as ‘raw’ data because data does not provide direct access to phenomena and because it always-already emerges as interpreted in one form or another. Consequently, scholars have called to study social media data as a product of particular historical, cultural and material configurations (Couldry & Van Dijck, 2015). Thus, sociality is not ‘simply whatever happens ‘on’ social media platforms’ (Couldry & Van Dijck, 2015, p. 2). Instead, platform data must be understood as a representation that is necessarily contested (Couldry & Van Dijck, 2015), and produced by a specific configuration of non-human and human elements.

The practitioners of positivist computational social science support their methods by pointing to the laboratory-like nature of the Internet (Lazer et al., 2009). I argue that this support needs to be turned on its head. The laboratory studies literature has established that the laboratory is far from whatever phenomena being studied but instead is a site of positive forces at work (Latour & Woolgar, 1986), a specific historical alignment of humans and non-humans for a particular goal. Thus, a significant amount of literature is dedicated to documenting the workings of such local contingency spaces (Knorr-Cetina, 1981; Latour & Woolgar, 1986; Traweek, 1992) and treating laboratories as agents of reconfiguration of the phenomenal field (Knorr-Cetina, 1995). Following such an approach, platforms should also be considered as agents of reconfiguration that improves upon the social order like laboratories improve upon the natural order (Knorr-Cetina, 1995) by bringing it home (Knorr-Cetina, 1999). To put it differently, platforms depend on the malleability of sociality to manufacture objects that yield revenue.

A direct comparison of laboratories of natural sciences and platforms of computational social sciences is helpful. The power of the laboratories stems from their ability to (1) eliminate the need to accommodate the object as it is, (2) where it is and (3) when it is (Knorr-Cetina, 1995). In other words, laboratories are infrastructures that bring nature home in order to create *workable* objects. Platforms bring sociality home in a similar manner:

1. Platforms exploit their intermediary position to place human sociality in a controlled environment. While sociality is always constructed, in the context of platforms the construction is controllable through design practices.
2. Platform infrastructures are set up to purify complex and messy human interaction into basic categories. Each platform encourages and discourages certain actions through their affordances that seem to converge over time among platforms (Bucher & Helmond, 2017).
3. Platform infrastructures can manipulate sociality. The infrastructures are set up in ways that can test different configurations with strategies like A/B testing (Keshavan, 2021) and manipulate sociality in the form of nudging (Wu et al., 2021).

Thus, platforms fulfill a variety of functions for computational social sciences that are fulfilled by the laboratory for natural sciences. However, while a traditional laboratory allows scientists to make certain decisions while still within the limits of the local contingency space, computational studies of the social do not afford such control to researchers. Fundamental decisions of data composition, quantification, and control (Knorr-Cetina, 1981) are often made by platforms prior to the study. The social emerges as an object as a result of these decisions.

My understanding of ‘the social’ here is similar to that in studies that have demonstrated the performativity of classifications (Bowker & Star, 1999), data (Fourcade & Healy, 2013, 2017), rankings (Espeland & Sauder, 2016), models (MacKenzie, 2006), standards (Scott, 1998), economic theories (Callon, 1998; Mitchell, 2008) and abstractions (Mitchell, 2003). For example, MacKenzie (2006) has described financial models as an engine that is productive of new sets of economic relations through models’ practical employment. Scott (1998), along with Foucault (2009), demonstrated that for the ‘population’ as the object of modern state to emerge, a whole range of methods of standardization and subsequent measurement had to be implemented that were never meant to perfectly depict societies but only to allow successful intervention when needed. An important difference that needs to be noted is that, in both Scott’s (1998) and MacKenzie’s (2006) cases, the metrics and the objects of measurement are relatively separate and thus, performativity of measurements are relatively weak. In the case of social media platforms, however, the relationship between the emerging social and the measures of platforms are tightly knit. This is because platforms are not merely facilitators (Gillespie, 2010), but are infrastructures of formatting (Koopman, 2019) that construct the social in alignment with corporate interests. In other words, the infrastructures of platforms are meant to manufacture a specific social: a standardized, homogeneous, orderly social such that it can be utilized for something else. Consequently, scholars have come to suggest that platforms like Facebook operate through a social logic of derivative (Arvidsson, 2016). Like financial derivatives, the social derivatives that platforms like Facebook produce cannot be reduced to what is supposed to be their underlying realities; in fact, such ‘derivatives operate by creating a virtual reality of relations and connections that need not have any ontological grounding’ (Arvidsson, 2016, p. 6). Thus, I understand the social of computational social science as the readily engageable, final result of standardization, formatting and abstraction mechanisms embedded in the infrastructures of platforms.

I propose that platforms constitute laboratories for computational social research. The data available for computational social research on social media is shaped by what platforms choose to record, measure, how and how frequently. Consequently, platforms provide data sciences with a domain (Ribes, 2020; Ribes et al., 2019) when it comes to social research. While there are, and rightly so, concerns about bias resulting from overrepresentation and underrepresentation of certain groups on platforms (e.g. Cesare et al., 2019; Hargittai, 2020), which can be performative (e.g. Brayne, 2021; O’Neil, 2016), the significance of platform-generated data for computational knowledge production is not only a matter of misrepresentation but also production. In other words, platforms manufacture a particular social through abstraction and standardization mechanisms embedded onto their infrastructure; formatting (Koopman, 2019) and production happen simultaneously. In doing so, platforms provide sociality as a domain for computational researchers: the social.^1^ Researchers may further standardize and abstract from platform data according to their needs, but the abstractions and standardizations of the platforms are the conditions of possibility of their work. Thus, the social of computational social research is an effect of such processes rather than a representation of a pre-existing sociality (Couldry & Van Dijck, 2015). Recognizing the full extent of performative effects of platforms opens up areas of questioning that go beyond criticizing platforms for being unrepresentative and invites us to theorize about what ‘misrepresentations’ of platforms do, how they organize and produce new realities.

## Methods

This article draws on field work conducted from 2022 to examine research practices of a university laboratory in North America to explore how platform-generated data shape computational social research. Over the course of 20 months, I spent following the work of the lab, it was organized into different teams focused on interrelated but different projects. Most projects were focused on language processing. Researchers often employed topic modeling^2^ and language modeling in their research, e.g., Latent Dirichlet Allocation (LDA), different types of word embedding^3^ and so forth. The lab is made up of approximately 15 researchers, with one serving as the director. The researchers are mostly graduate students.

Projects of the lab ranged from tracking public discourse through topic modeling to creating experimental media platforms. The most prominent project at the time of fieldwork was the project that tracked discourse online using Twitter data and how it changed in response to news, statements of public officials with regards to the COVID-19 pandemic.

I started my field work by interviewing Jay, the director of the lab, and observing weekly and biweekly online meetings of the laboratory. Additionally, I conducted interviews with lab members and researchers who were not affiliated with the laboratory, resulting in a total of 15 in-depth interviews with 10 interviewees. Observations of meetings began in June 2022 and encompassed approximately 50 hours of observing lab members. Because of the lingering Covid-19 pandemic, all observed meetings were conducted through videoconferencing. While the virtual nature of these meetings provided easier access, it also meant that there were fewer opportunities for informal conversations with lab researchers. The research material includes the accounts of research and data practices obtained during interviews with lab researchers, as well as other researchers in the same field who are not associated with the lab, in addition to observations of meetings and other relevant events.

## Findings

In my observations and interviews, data quality was a prominent topic that was expressed by all researchers regardless of their research problem and methods. Specifically, the researchers expressed a strong desire for ‘good’ data. For my informants, two important requirements pertaining to data make their work possible: standardization and association. A collection of information that is not standardized and/or where relationships between entities are not made explicit is ‘bad data’, data that is unusable with computational methods. Standardizing sociality and associating digital entities are also two core processes that platforms need, and their infrastructures perform. In what follows, I discuss the roles of standardized and associated data provided by platforms for my informants’ work.

### Standardizing

The outsized role of standardization for my informants’ work can be illustrated through the following conversation with Ronald, who was working on studying public discourse. During our interview, Ronald explained to me that the lab had gathered data for the project from newspapers, speeches of public figures, journals, and Twitter. But they found out that different data sources come with different problems. The most significant problem was that data from different sources have structural differences that could easily be mistaken for interesting findings. The method Ronald was using for this project was topic modeling, or more specifically Latent Dirichlet Allocation (LDA, see Jelodar et al., 2018), where the model generates topics on the basis of words that may or may not co-occur and produces clusters of words that it deems representative of the document analyzed. This means, Ronald told me, that it is particularly prone to generate clusters on the basis of what might be structural differences rather than interesting findings. Comparing Twitter discourse with press releases and speeches of public figures while at the same time walking me through some graphs, Ronald said:

So, collecting Twitter data is fairly simple, but the other things they tried to collect were press releases … articles and journals, and stuff like that. … We weren’t able to do this what I’m showing here on that type of stuff. Because … all that stuff is so structurally different that when you stick it in the algorithm, all the algorithm … spits back is those structural differences. … If you, if you grab 100 papers about (Covid) … and maybe mix in 100 other random articles, … you’re going to start seeing not the separation between Covid and non-Covid, you’re going to start seeing the differences in the papers … like you know, ‘Oh, this cluster is one published by *New York Times* and this cluster is …’ just because, you know, the words are that different.

Without a common ground that ensures that all data is generated in a standard way, data cannot be analyzed to come up with different clusters of words that serve as topics. In other words, according to Ronald, when there are large structural differences that may be arising from, for example, different styles of writing or different editors with different tastes, all clusters that the model can generate will be based on those differences. This makes it very difficult to produce knowledge about social phenomena, because they occur all over the place and are not suitable for analysis as they are. All scientific knowledge production depends on preconstruction of the object of analysis in controlled environments (Knorr-Cetina, 1981). Social scientific knowledge production is no exception. For social phenomena to be fit for study using computational methods, they must be constructed in a predetermined way in predetermined environments. In other words, in a laboratory.

Computational approaches require computational objects, regardless of whether they are prepared or collected as such by the researchers. Twitter’s API provides the researchers with these objects. The tweet object for Ronald’s project was the basic unit of analysis provided by the platform in an already packaged fashion. The standardization, however, does not end here. Ronald mentioned that one of the advantages of working with data from only one platform is that digital objects on the platform share the same restrictions. For instance, all tweets on Twitter have a character limit. But in addition, says Ronald, Twitter standardizes how people talk and interact:

[There is] talk about you know left Twitter and right Twitter, but … there’s still a common sort of culture on how people write … limited by the … platform …. How people retweet and stuff like that.

According to Ronald, the boundaries that Twitter draws around the domain of possible actions/interactions on the platform results in a commonality in practices which, in turn, ensures consistency in the data generated. This often manifests itself in the most mundane ways, such as through spell checkers. Ronald says:

[Twitter is] homogeneous structurally. … [A]ll of the tweets have the same restrictions. They’re all working with the same spell checker. … So, there’s less structural issues that appear in the things, and if there’s one thing that AI is really good at, it’s finding structural problems with your data.

LDA, the approach that Ronald uses to make supposedly existing latent topics in documents manifest, relies on the assumption that documents are bags of words, and processes them on the basis of the relative frequencies of each word in different documents. This approach would be impossible for Ronald to use if the language on Twitter was not standardized to some degree. The models Ronald uses cannot reliably tell whether ‘resident’ is a word of its own or a misspelled ‘president’. This is a mundane, but still significant problem for Ronald, he says, talking about insurance forms that are filled manually by people, that ‘nobody knows how to … spell Mitsubishi. Nobody does.’

However, the production of the social through standards also has its downsides, as the standards of platforms differ. This means that it is difficult for researchers to combine data from different platforms when using certain methods. Pierre, a member of the same lab as Jay and Ronald, working on a similar project to Ronald’s, said:

So, if … 85 or 90% of your data set consists of tweets and then you inject 10 percent of other platforms, at the end, everything gets mixed up. So, at the end of it, you will not be able to understand what comes from what part. … What does it do within the data set? So maybe it doesn’t do anything, maybe just worsens the quality of the data.

By worsening the quality of the data, Pierre is referring to the mixing up of standards that exist on different platforms. Because Twitter has a particular way of enabling users to express themselves bounded by the character limit, features, and the Tweet form in general, injecting data from other platforms where people may express themselves completely differently, in longer formats for instance, may worsen the quality of data according to Pierre. He says:

Twitter has a style, a stylistic way of expressing itself because of social habits, for sure. Very few people go to Twitter and it’s like, I’m just going to write my essay on Twitter and distribute on different tweets. Tweets are a form of expression in itself. … So, … whenever you have unsupervised language model learning … when you put a substantial part of extra material coming from forums and blogs where people express themselves in a completely different way, the model might not work as well because, again, you have two different forms of expression completely different and in different sizes.

As in the previous example about structural differences between datasets, mixing data from platforms with different approaches to modeling human sociality, again, introduces a structural difference within the dataset that may worsen the quality of data and thus, quality of the resulting model. In fact, Troy, a computational researcher not associated with the lab but who worked with Reddit and Twitter data, told me when I asked him about whether a model that is trained on Twitter data works on Reddit or vice versa:

No, it doesn’t work very well. … I mean, they’re very … they’re like different languages altogether, … there is no similarity between Twitter and Reddit, it’s completely different worlds. … Twitter posts are by nature less than 280 characters; Reddit posts are sometimes like as long as … entire book chapters. There are other differences, … Twitter is person centric, meaning that in on Twitter you basically have each person representing their own profile. Reddit is not person centric; Reddit is topic centric … where people basically go and post in special interest forums called subreddits … there are all these differences between Twitter and Reddit in terms of how the social network works.

For Troy, as for Pierre, the forms of different platforms can be so different from each other that they can be incommensurable to the point that a computational model trained on data from one platform may not work very well on data from another one.

### Associating

The second epistemological aspect of platform data for computational work is associating. For computational modeling work to be possible, there needs to be a platform that establishes different relations between different properties of different elements and standardizes them in forms that make them fungible. Unassociated data is ‘bad data’, according to the researchers. Ronald, who also works as a full-time engineer, often comes across ‘bad data’.

Another good example of this I find in the financial world quite a bit, where people will use a unique identifier on the person and a unique identifier on a loan. But frequently they forget to cross reference the two … Let’s say I have an accounting system and I care about whether or not someone’s paid back my loan. … I want to … do a machine learning model about whether someone has paid back the loan and if I can’t correlate the original application with the loan then I could have all this data and it’s completely worthless.

Though this might seem like a trivial example, it illustrates a larger point: Bad data is data where the relationships between properties of different elements are not made explicit. In other words, to follow Ronald’s example, unless the unique identifier of a person and the identifier of their loan are connected, regardless of how much of such data you have, it is unusable. Another example of this is issues with time zones, Ronald explained:

[I]f it’s a case like let’s say time zones, sometimes you can correct it. … sometimes I can infer ‘OK, they’re doing local time. Their local time is this. I’m going to correct it.’ Sometimes you can, you know, fix spelling mistakes. Uhm, but other times you really can’t do anything. You just kind of got to discard it because it’s not useful.

**Figure 1.**
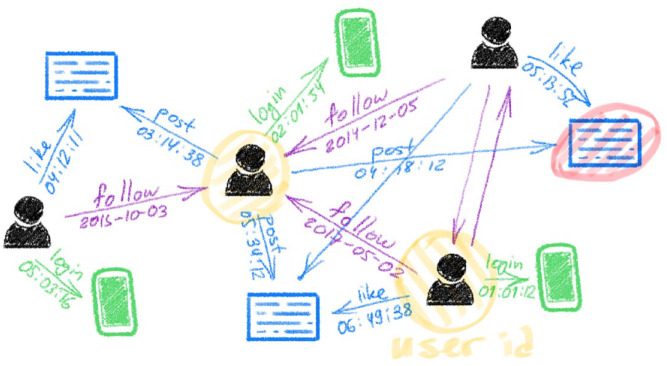
Twitter as graphable associations, reproduced from Bronstein (2020).

Data, which are often understood in terms of ‘information that something happened’ (Srnicek, 2016, p. 20), are meaningless unless they are ‘information that something happened in association with something else’. This difference is crucial to understand the first function of platforms in making computational work possible: Platforms allow emergence of entities always-already in association. In other words, data are not a raw material that already exist outside that needs to be extracted and refined like oil (Srnicek, 2016). They are generated in relation to each other in an environment much like laboratories. The social graph of Facebook is not a representation of how people are connected in supposed life processes, but it is an abstract space of virtual sociality (Arvidsson, 2016). This space is a space of preconfigured relations: Users and posts on Facebook, for instance, are objects that can have edges (relations/interactions) with other objects (Arvidsson, 2016). However, it is not a space where an object can exist without any sort of connection to another. Michael Bronstein, head of Graph Learning Research at Twitter, represents Twitter in Figure 1.

Each object on the platform, a post, or a user, emerges in connection with some other through different types of ‘edges’ e.g., a like, a follow, a retweet and so forth. Thus, data, when they emerge on platforms, emerge always-already connected to one another because otherwise production of metrics and thus, social media data as an asset (Birch et al., 2021) is not possible. This is a highly concrete process. Looking at sample Twitter API (Application Programming Interface) responses one can see fields that exist for each tweet such as in_reply_to_user_id, referenced_tweets, id, author_id, metrics and so forth (Twitter, n.d.). Ronald’s other example of having different time zones in different databases, again, is not an issue because that is also delegated to the platform; it does not matter what time a person posts a tweet in their local time because the data generated is recorded centrally. For instance, Twitter’s API, when one queries for tweets, returns data that contains a field that is the exact timestamp in Coordinated Universal Time (UTC) and may contain another field (if the owner of the account configured their timezone on the platform), which represents the difference between an account’s timezone and UTC in seconds (Twitter, n.d.). Thus, ambiguities that may be introduced by users and their locality are surpassed through the socio-technical infrastructure of Twitter.

This always-already associated nature of different nodes in data generated on platforms solves the association problem for the researchers. This is because platforms take care of much of the work of associating data. Ronald’s example of people putting their unique IDs incorrectly in different databases that later need to be merged is not likely to happen on platforms because such work is delegated to the infrastructure. On platforms, the boundaries of the domain of possible actions/interactions are drawn clearly: one can tweet, retweet, mention, like, view etc. Each of those actions are associated in a central database where there is no tweet without an author, a followed account without a follower, no question of whether two different tweets belong to the same account or not and no question about time zone ambiguities. This is similar to mundane laboratory work in natural sciences involving labeling, recording, and tracking samples, work that later gets omitted from the final product (Latour & Woolgar, 1986). In the case of computational social research this work is delegated to socio-technical infrastructures of platforms.

### Frictions: Different platforms for different questions

Although the researchers are aware that data from platforms may not be enough to speak to the larger population, there are advantages to treating platforms like Twitter as surrogates for public discourse because they make computational social research possible by producing a standardized form of social and associating the entities within following rules that are more or less stable over time. For instance, Ronald, talking about the effects platform designs may have on the discourse, explained to me:

I don’t know if I would go as far to say that media is the message. But it absolutely has an effect on the message. … one advantage we have from a computational perspective is that … it’s a knowable bias. Like certain biases … we have to discover. But the one thing about computers, specifically things like Twitter, is we know their rules. You know, I don’t know exactly how their algorithm works, but I know that it is limited to 140 tweets [sic]. I know you reply to individual people. … we can figure out what the rules are, and we can mathematically model those rules. … so, it actually becomes a little easier to model bias inside these things simply because we know the bias is considered mostly consistent. Twitter changes, … the platforms change. They’re consistent enough over a period of time. You know, Twitter isn’t redeveloping their algorithm every two hours.

According to Ronald, the fact that discourse on platforms occurs on a controlled and stable setting makes it, theoretically, possible for researchers to account for the bias that is introduced by the design of platforms. While individual platforms may be biased in one way or another, those biases are knowable and can be accounted for by the researchers. Ronald explained to me that this could also make it possible compare discourses on different platforms given that they account for the different biases introduced by each platform. However, this seems to be a hypothesis for the time being, as I have found that researchers prefer to work with different platforms depending on circumstances; not every platform is suitable for every computational approach or research question.

As not all platforms employ the same standardization methods and make the same design and standardization decisions, the data they generate may be more suitable for different types of questions, methods, and analyses. For instance, depending on the research question and the computational methods utilized for analysis, different features of Twitter may prove to be advantages or obstacles for researchers.

Pierre’s project relies on word embedding i.e., transformation of words into vectors in high-dimensional space to capture the relationships between words. While it can work with relatively unstructured text, word embedding thrives on relatively structured, rich text according to him. Pierre has been using word embedding in order to analyze the transformation of a discourse around a scandal that mostly occurred on Twitter over time by analyzing what he calls ‘episodes’, i.e., time frames during which certain words tend to occur in a stable semantic relation to other words. For instance, in an imaginary discourse around ‘capital’, if the word ‘capital’ is closely related to ‘exploitation’ in the first weeks of the discourse and later it relates more closely to ‘innovation’, would be considered two distinct episodes where the semantic relation of the word ‘capital’ to other words has changed. When Pierre needed to apply word embedding to Twitter data, he encountered several difficulties. He explained his experience with Twitter to me:

[I]t’s a nightmare to work with. … You have short sentences, a lot of hashtags, that’s stuff that you have to remove. … Twitter is so fragmented that you need to make a choice at the very beginning. … how am I going to clean or am I going to treat the text? Do I want to keep the hashtags? Do I want to remove the hashtags?

Pierre explains that in his project, hashtags were a huge problem because they continuously interrupt the sentences. Pierre explained that word embedding requires sentences to be somewhat syntactically correct so that it can infer which words are nouns, verbs etc. If the sentences are continuously disrupted by hashtags, it makes it harder for the model to learn. But hashtags are not always obstacles:

If you’re working on a social network analysis, you want to have hashtags. You want to have a second layer of nodes besides, for example, the authors. You can choose your nodes. Nodes can be topics. Nodes can be hashtags themselves. I absolutely know that it was extremely beneficial for Ronald … to work on topic modeling. … The problem of word embedding is that they work as natural language processing environments. And hashtags most of the time are ungrammatical. So, meaning that not just they’re not well formed grammatically, but … they occupy a space that should be occupied by that type of word.

In other words, while hashtags are useful in social network analyses or when one is doing topic modeling, like Ronald, for Pierre they were obstacles because the methodology he was using required a particular type of expression; the more grammatical the better. Further, word embedding, Pierre says, works better when the material is longer, thus, the character limit imposed by Twitter at the time of data collection was also a problem for Pierre. For instance, when I asked him about the alternative platforms he would like to work with, he replied:

So, what I would like to work for or on … something that really, really has a lot of available text and I really like to work on reviews. I really like to work on Metacritic, for example. … … Most of their reviews are well written. Not only that, but you have multiple choices of language because again, we’re just focusing on the standard, which is English, but luckily for us, that’s not the only language available in the world. So, you can actually deal with multiple languages. … I think … Metacritic would be interesting or even IMDB since Amazon bought it off. So, you can just check how things have changed, for example.

Compared to Twitter, Metacritic, he says, contains well-written reviews that easier to work with using the methods he works with. In contrast:

Twitter has this obsession for being sharp and clear cut, it has an expression … a Twitter lingo, which goes even into condensing or shortening words themselves. So, you … might find that ‘thanks’ is a little more time consuming to write than just ‘thx’ like that, which again, linguistically is a problem. The problem is solved or is attenuated by the volume again, by the fact that on a larger scale, the larger the pool of the intake you have, the less likely those sorts of things, those sorts of bits of language here and there are to be less relevant. … I think the majority of tweets and the majority, I think the 50% plus 1 are somehow grammatical …. And where that doesn’t happen, you as a text curator have to make a decision on what to do with the others, keep them in [or] just erase them altogether. This is not a problem that you would have with other platforms. You would just do some basic text cleaning, eliminate just the weirdness of some words or the typos or maybe not even that because if the volume is so large, it doesn’t really matter and that would be it. So, you don’t make many decisions on other platforms. In Twitter, you’d really have to make a decision of what to do with the language that you’ve been working with, which of course alters the nature of the language. So, it’s not 100% Twitter anymore.

For Pierre, Twitter is unique in the way that it has certain dominant ways of expression that causes problems for types of analyses Pierre does and makes the preparation/data cleaning prior to the modeling much harder where one has to make decisions as to how to prepare for word embedding.

The ungrammatical nature of hashtags, the character limit, and the dominant ways of expression on Twitter, while huge problems for Pierre, were not of much importance for Ronald’s project. Ronald’s project, while similar in research question, used topic modeling, and specifically LDA, instead of word embedding. Hashtags were a blessing for Ronald because they enabled data filtering from Twitter, and curation of his dataset. In other words, if you want to curate a dataset that is supposed to be about, for instance, how people in Barcelona feel about the Spanish National Anthem, the hashtags #barcelona #MarchaReal work to filter the tweets, so that you can get only tweets that are supposedly relevant to the research question. Not only not an obstacle, but hashtags for Ronald were also a necessity so that he could filter out the conversation related to his research question. Similarly, when talking about problems with working with Twitter data, Ronald never mentioned the character limit or the dominant styles of expression on Twitter as problems. In fact, they were beneficial for him as elaborated above because above all else his project needed consistency.

Noah, another researcher outside the lab, echoed these sentiments. One of his projects was aimed at extracting proxies for mental health problems from tweets. To do so, he created two cohorts of people, where one group expressed at some point on Twitter that they had been diagnosed with a mental illness while the other cohort did not. When I asked why he had decided to use Twitter data, he explained to me that, in terms of ease of access and availability they had two options, Reddit and Twitter. However, his group had decided to use Twitter instead of Reddit particularly because of the differences between how these two platforms are designed to work:

So, we talked about other platforms. … So, for instance, one of the ideas would be to use Reddit but then maybe you don’t have the concept of timelines since we were interested in looking at longitudinal data for specific users. We turned to Twitter because that longitudinal aspect of it was one the main reasons to investigate it. … So, if you’re interested in the activity of one specific user in Reddit that’s not directly attainable right? … If you want to use Reddit data, it’s topical by nature so it’s all pertained to specific topics and not necessarily every message that one particular user has posted in its history on Reddit.

Noah explains here that Reddit is designed to work around topics and that there is no easy way of tracking a particular user over time to study their mental health through their posts and comments while Twitter allows for this kind of an approach. Thus, studies that would not be possible or significantly harder with one platform can be a lot easier with another. In fact, Noah told me that for any project that requires longitudinal data he always turns to Twitter. When I asked about whether the available data plays a role in the research questions they ask, Noah replied:

Well, it definitely shapes the questions that you’re able to answer. In the sense that we have had discussions about possible avenues to pursue where we just have to where we ended up saying, ‘Well, yeah, it would be very cool if we could do this but there’s just no way that we are going to get this particular data’ … Those are the limitations you have to work with, but I think it’s important to not start thinking from those limitations when you’re … What tends to happen … is that I’m discussing ideas or discussing a current analysis of a particular data set that we already have, or some ideas that I have, and then you start hypothesizing what other possibilities you would have or wouldn’t it be interesting if we could also measure that from this particular set of individuals … what happens if we would try to measure this on a different platform? Is that even possible, if yes, what could we measure?

As evident from Noah’s response, the ready availability of pre-packaged and standardized data from platforms, influenced by their specific standardization processes, has a profound impact on the types of questions researchers are inclined to ask and the methods they can employ to address them. In fact, a crucial research aspect, such as determining what to measure and how to measure it, appears to be considerably influenced by platforms rather than being solely determined by researchers themselves.

Finally, it is important to note that how the researchers imagine the platforms also plays a role in the selection of platforms for data curation. Although he was hesitant to do so, not being an adept user of these platforms, Pierre compared Facebook and Twitter in terms of their usefulness for different kinds of research:

I think Facebook is more subject centered. I think the person on Facebook … because of the page, it’s more like proposing or sending out the image of themselves that they would like to portray. Twitter is more topic oriented. … Again, these are more like sensation. I really shouldn’t. … So, I think this is the main difference between the two. And therefore, the first one being more the subjective side of things, it allows for introspective, although a little bit cringe, … but introspective posts and text.

Although it was not the case in this project, Pierre’s remarks indicate that the researchers have different ideas about the functions of each social media platform and depending on the questions that they have, they may pick and choose the platform they will work with based on these ideas. In other words, the ideas of researchers about different social media platforms as well as the way the platforms organize the social may also determine which platform they work with besides the material constraints like the ease of access to the platform. These imaginaries, however, seem to change based on the researcher. For instance, Noah above expressed that he considers Twitter to be subject-centered and Reddit to be topic-centered, while Pierre expressed the opposite about Twitter.

## Discussion

Both computational researchers and platforms share the need to delineate, standardize and measure. Computational researchers need to standardize their data so that the input features to their models have a consistent format and that the features align with each other. Platforms need to delineate, standardize and measure because they generate revenue by constructing abstractions, such as ‘users’, that serve as metrics (Birch et al., 2021). This shared objective creates a symbiotic relationship where social media platforms effectively function as laboratories for computational social research: Platforms become spaces of standardization, association, and abstraction where indeterminate and messy sociality is carved out selectively and transformed into an interested, structured research object.

As scientific laboratories tame the unruliness of nature (Knorr-Cetina, 1981), platforms tame the uncertainty and messiness of sociality. First, in order to create assets (Birch et al., 2021) to generate revenue, platforms are designed to manufacture a standardized and associated social which results in the demarcation of the social as an object. Platforms enable the manufacture of data about the social in controlled environments and, in the case of platforms like Reddit and Twitter, make the data accessible in specific, standardized formats for computational use. These are accomplished through embedding abstraction mechanisms onto platform infrastructures as features (retweets, likes, etc.). Such features both enable the production of metrics for the platforms and the emergence of human interaction always-already in formats that lend themselves to creation of metrics. Through them the social is manufactured as a standing-reserve (Heidegger, 1977) or a resource that is ready to be used (Busch, 2019).

Second, platform infrastructures perform hidden menial, technical work that needs to be done in conventional laboratories to make facts speak for themselves. Menial tasks like labeling samples, storing them, and recording the samples’ relation to each other are all delegated to the socio-technical infrastructures of the platforms and made invisible. Like lab technicians (Shapin & Schaffer, 2011) or microwork (Irani, 2015), this invisible labor of the platform infrastructures takes care of the messiness of the social and helps create the image of platforms as uncomplicated and straightforward representations of sociality rather than a fabricated ‘social’, and, in the process, bolsters the cultural authority of computational researchers and data sciences as experts for social problems.

Third, platforms are prospecting (Slota et al., 2020) agents. Prospecting in data sciences is the work that needs to be done prior to any kind of analysis or visualization to transform a part of the world into a computationally accessible format. In producing the social as a standardized, homogeneous, associated entity, platforms perform this function and manufacture the social as an always-already engageable domain for computational sciences. Such readiness bolsters the claims of data and computational sciences to be metadisciplines, disciplines without a domain but a relevance for all domains (Ribes et al., 2019; Slota et al., 2020)

Fourth, platforms enable computational researchers to narrow their truth claims and make making such claims easier. When researchers choose to work with data from only one platform, for the reasons discussed above, this allows them to reference their dataset as something definite. For instance, Pierre explained to me when talking about why it makes things harder to mix in data from multiple platforms:

Yeah, because again, you want also to have your data set to be clear cut in a way. You want to be able to reference as a data set as something that you can manage, but you can just point to. … So, I think, since we cannot go back from the results to the dataset, having the data set explicitly, again, defined and knowing that everything that we produce comes from Twitter 100% because that’s the only dataset we would be taking as a source. I think that would make more sense.

Pierre highlights the importance of working exclusively with data from a single platform, as it enables referring to the dataset as ‘clear-cut’. This approach strengthens the credibility of findings when presented to the scientific community. It not only allows researchers to make truth claims more easily, but it also ensures the broader scientific community that the research is reproducible to a certain degree. In essence, platforms act as the local contingency space for all participating laboratories, facilitating delineation of research boundaries and promoting streamlined discussions of research reproduction (Knorr-Cetina, 1981, p. 39).

If ‘nature is not to be found in the laboratory’ (Knorr-Cetina, 1981, p. 4), society is not found in the platforms of computational social research either. In computational social research, the social is not a pre-existing entity that can be captured or extracted. The abstraction mechanisms employed by platforms are productive, actively shaping and enforcing the creation of specific types of ‘the social’ which varies with different strategies platforms employ. We need to understand ‘the social’ in computational research as constituted by platforms rather than a representation of pre-existing sociality, as an engine rather than a camera (MacKenzie, 2006). Of course, social science, computational or otherwise, always operates on and through constructions. The particularity of computational social science in this regard is the displacement of the construction process of the research object and the delegation of the latter to infrastructures beyond the control of researchers themselves.

However, unlike scientific laboratories, platforms’ infrastructures are shaped by concerns other than purely scientific ones. The same abstraction mechanisms that provide well-structured data to computational researchers are what platforms use to generate value. These mechanisms are products of decisions of social media companies: how to design the platform infrastructure, which metrics to produce, and which kinds of sociality to allow and disallow or encourage and discourage (Grosser, 2014). The theoretical and methodological aspects of addressing social aspects, selecting data parameters, determining units, and gathering data are all influenced by the platform itself. Consequently, the data generated is aligned with the platform’s preferences and priorities and a part of the scientific agency of researchers is stripped away. Thus, the link between contemporary epistemologies of computational social research and platforms lies in the ways features, metrics, and standards are produced in accordance with their practical potential for commercial applications.

## Conclusion: Digital capitalism, epistemology, and the power of data scientists

The main concern of this article was to understand platforms as laboratories, infrastructures that manufacture the social for computational social science. I have found that platforms play a significant role in computational social research, because they take care of much of the selective carving out of the social and its transformation to a research domain, in turn shaping the questions asked and the methods and approaches used in research. Platform data, for this kind of computational social research, fulfills several functions: It forms their research object materially, provides the local contingency space, does prospecting work, and allows researchers to delegate mundane laboratory work. Further, diverse platform designs and features influence the research questions the researchers can ask and the research methods they employ.

The frictions that researchers encounter across data from different platforms also underline the limits of the extraction metaphor undergirding theories of digital capitalism and invite us to consider more fully how platforms’ sociotechnical infrastructures produce different kinds of data that can be capitalized on in different ways. Platforms’ concern with producing metrics through features, while often advantageous, can also lead to frictions for researchers when working with social media data. This is a result of a paradox for all social media platforms (van Dijck, 2013): They promise to deliver accurate representations of the social to their advertisers, but their business model relies on them managing, governing and engineering the social in order to generate revenue. Then, far from being a representation of the social in itself, the social on platforms is generated on a socio-technical infrastructure that aims to transform the social into a computational and profitable object. In addition to the concerns raised by scholars about the involvement of Big Tech in academic research and public use of AI (Abdalla & Abdalla, 2021; Birhane et al., 2022; Phan et al., 2022; Stinson et al., 2020), digital capitalism exerts its influence on computational social research through its control over the online social realm but also through its manufacturing of the social based on their concern with capitalization. In doing so, platforms influence academic research at an epistemological level as well, through producing the research domain, shaping the questions asked, data available and methods used.

Finally, the outsized role of standardized and associated data for computational social research also invites us to reconsider recent claims of computer and data sciences to being metadisciplines (Ribes, 2020; Ribes et al., 2019; Slota et al., 2020), i.e., sciences of everything that can be applied to any area regardless of qualitative differences that may exist between objects of study. However, we have seen that different data science methods require different kinds of data and different styles of standardization. Prospecting is a precondition of data sciences’ being able to engage with this or that ‘worldly’ (Slota et al., 2020) domain and it undergirds the claim to universality of data sciences. For computational social researchers that work with social problems, social media platforms as socio-technical infrastructures that act as prospectors; they produce the social as a domain that can be engaged with data science methods. Then, data and computer science methods’ seeming versatility is not an inherent quality of them, but rather it is achieved through the infrastructures of social media that take care much of the prospecting work that needs to be done prior to any kind of analysis or visualization. Thus, these disciplines’ current epistemic and cultural authority results, in part, from digital capitalism’s tendency to produce objects that are compatible with the epistemologies of these disciplines. The automation of the production of the social as a standardized domain appears to undergird the claims of data sciences to be universal and domainless by hiding the work of prospecting and production of the social. However, once we consider social media platforms as laboratories and their infrastructures as productive, we can see that computer and data sciences methodologies are not without a domain and social media data is not without interpretation. Instead, digital capitalism tends to favor these methodologies and thus automates the production of the social on platforms in ways that are compatible with them i.e., it produces pre-interpreted, standardized, and associated objects of analysis. This, then, suggests that the contemporary authority of data science as ‘science of everything’ may be scaffolded by digital capitalism. Thus, future research on the effects of social media data on computational knowledge production should problematize the socio-technical infrastructures of platforms as historical, material assemblages that necessitate examination of their effects. Comparative studies are essential to assess whether reliance on platforms in social research skews emphasis on specific social practices and meanings.

## Notes

1. Latour (2005) argues that the social should not be thought of as a domain of its own but as a dynamic of network of relationships does not hold for the social of contemporary computational social research. The controlled nature of platforms, while still a network of humans, non-humans, and ideas, is a strategy to reduce or eliminate the heterogeneity that Latour (2005) uses to deconstruct the social.

2. Topic modeling is the generation of topics on the basis of words that may or may not co-occur and produces clusters of words that it deems representative of the document analyzed (Jelodar et al., 2018).

3. Word embedding is a technique in natural language processing where words or phrases from a vocabulary are mapped to vectors in a low-dimensional space, relative to the size of the vocabulary. This enables the model to capture the context of a word in a document, its semantic and syntactic similarity, and its relationship with other words (Ghannay et al., 2016).
